# Unsupervised Variational-Autoencoder-Based Analysis of Morphological Representations in Magnetic-Nanoparticle-Treated Macrophages

**DOI:** 10.3390/bioengineering13010076

**Published:** 2026-01-09

**Authors:** Su-Yeon Hwang, Tae-Il Kang, Hyeon-Seo Kim, Seokmin Hong, Jong-Oh Park, Byungjeon Kang

**Affiliations:** 1 Graduate School of Data Science, Chonnam National University, Gwangju 61186, Republic of Korea; hsy1076@gmail.com (S.-Y.H.); kimhs9574@jnu.ac.kr (H.-S.K.); 2 Korea Institute of Medical Microrobotics, Gwangju 61011, Republic of Korea; kti@kimiro.re.kr (T.-I.K.); seokmin.hong@kimiro.re.kr (S.H.)

**Keywords:** magnetic nanoparticles, macrophages, unsupervised learning, variational autoencoder, morphological analysis

## Abstract

Magnetic nanoparticles (MNPs) are widely applied in biomedicine, including bioimaging, drug delivery, and cell tracking. As central mediators of immune surveillance, macrophages phagocytize foreign substances, rendering their interactions with MNPs particularly consequential. During MNP uptake, macrophages undergo cytoplasmic remodeling that can lead to morphological alterations. Although prior studies have predominantly focused on MNP uptake efficiency and cytotoxicity, systematic quantitative assessments of macrophage morphological alterations following MNP treatment remain scarce. In this study, phase-contrast microscopy images of macrophages before and after MNP treatment were analyzed using unsupervised variational autoencoder (VAE)-based frameworks. Specifically, the β-VAE, β-total correlation VAE, and multi-encoder VAE frameworks were employed to extract latent representations of cellular morphology. The analysis revealed that MNP-treated macrophages exhibited pronounced structural alterations, including membrane expansion, central density shifts, and shape distortions. These findings were further substantiated through quantitative evaluations, including effect size analysis, kernel density estimation, latent traversal, and difference mapping. Collectively, these results demonstrate that VAE-based unsupervised learning provides a robust framework for detecting subtle morphological responses of macrophages to nanoparticle exposure and highlights its broader applicability across varied cell types, treatment conditions, and imaging platforms.

## 1. Introduction

Recent advances in artificial intelligence have underscored the importance of representation learning in effectively interpreting and organizing complex, high-dimensional datasets [1]. In particular, within the biomedical imaging field, unsupervised and self-supervised approaches have emerged as compelling solutions to address the limitations of labeled data [2,3,4]. However, data volumes across medical imaging, biological microscopy, and digital pathology are rapidly growing, and the implementation of supervised learning remains constrained by the substantial time and cost required to produce accurate annotations [5]. These limitations are similarly evident in cell imaging, where manual labeling is both labor-intensive and frequently inconsistent [6,7,8]. Consequently, unsupervised learning techniques, capable of extracting underlying representations without manual annotation, have emerged as a powerful and practical alternative [9,10].

Macrophages serve as central effectors of the innate immune system and exhibit notable plasticity as they adapt their functional states and morphology in response to external stimuli [11,12,13]. Their polarization into pro-inflammatory (M1) and anti-inflammatory (M2) phenotypes involves both biochemical changes and morphologically distinct features that are visually discernible [14,15]. Recent studies in nanomedicine further reveal that, owing to their structural heterogeneity, macrophages function as crucial regulators within tumor microenvironments and in immune regulation [16,17].

Magnetic nanoparticles (MNPs) are extensively employed in biomedicine for targeted drug delivery, cellular imaging, and immune modulation [18]. This widespread use is attributed to their responsiveness to external magnetic fields [19,20]. Furthermore, surface functionalization can improve their targeting specificity and expand their utility in therapeutic applications [21]. Supporting this, experimental studies have confirmed that macrophages internalize dextran-coated MNPs, leading to functional effects such as oxidative stress regulation and cytokine modulation [22]. Beyond these applications, MNPs have also been employed in targeted therapies, image-guided diagnostics, and tumor microenvironment modulation [23]. Transcriptomic and imaging analyses have further revealed that MNPs influence immune responses [24]. Nevertheless, despite these broad applications, the effects of MNPs on immune cell morphology, particularly that of macrophages, remain insufficiently investigated [25].

Conventionally, morphological changes in cells have been analyzed using supervised learning models that rely on expert-provided annotations [26]. However, these models are constrained by subjectivity, high time costs, and limited ability to detect subtle morphological variations [27]. Moreover, the structural and functional heterogeneity of macrophages, particularly under external stimuli such as polarization, hinders consistent labeling. Consequently, supervised classification sometimes fails to capture gradual morphological transitions and tends to oversimplify complex phenotypic heterogeneity [28]. In response, recent studies have emphasized the need for automated, label-free approaches for tracking dynamic cellular states [7,8].

In this context, unsupervised deep learning methods, particularly variational autoencoders (VAEs), have been explored for their ability to learn latent representations from unlabeled image data [29]. These models effectively extract intrinsic morphological structures and facilitate the quantification and visualization of phenotypic variation in a data-driven, label-free manner. Given these advantages, VAE-based frameworks have recently been applied to biomedical image analysis tasks such as drug discovery and cellular profiling [30,31].

Against this background, the present study developed an unsupervised learning pipeline to analyze morphological changes in macrophages induced by MNP treatment. This pipeline was designed based on phase-contrast microscopy, which serves as a non-invasive and label-free imaging approach. Building on this imaging platform, the analysis focused on multiple VAE-based models, namely β-VAE, β-total correlation VAE (TCVAE), and multi-encoder VAE (ME-VAE), with respect to their disentanglement capacity and interpretability [32,33,34,35]. Before model training, the images were preprocessed using grayscale conversion, contrast limited adaptive histogram equalization (CLAHE), sharpening, resizing, and min–max normalization. Following this preprocessing, model performance was assessed by computing Cohen’s *d* effect size for each latent dimension and by visualizing phenotypic differences through latent traversal and structural similarity index measure (SSIM)-based difference maps [36,37,38]. Notably, compared to traditional qualitative imaging techniques such as fluorescence or electron microscopy, this unsupervised approach provides a quantitative, scalable framework for assessing morphological phenotypes using only phase-contrast images [39,40]. The principal contributions of this study are as follows:

• VAE-based models effectively identify macrophage morphological changes induced by MNPs within an unsupervised framework.
• An integrated evaluation framework is introduced, combining statistical effect size analysis with interpretable visualizations.

**Figure 1:**
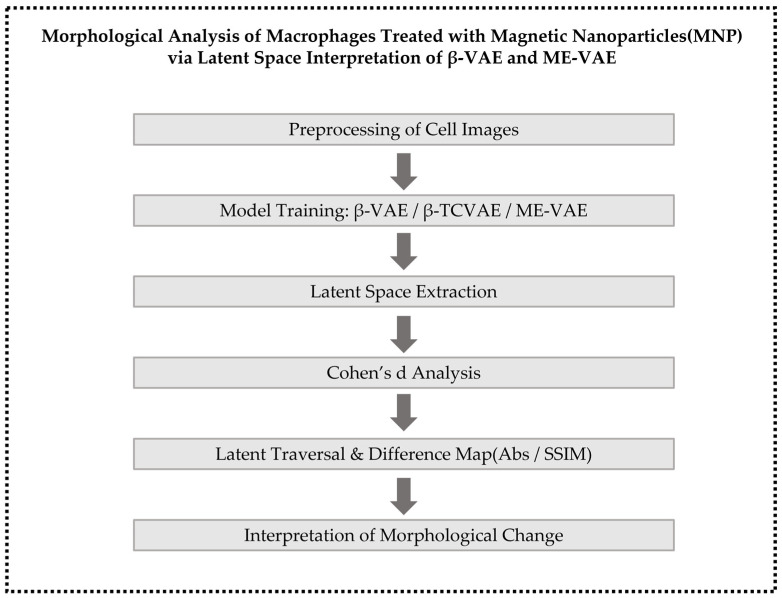
Workflow of the unsupervised learning pipeline for macrophage morphology analysis.

Overall, this study bridges artificial intelligence and nanomedicine by offering a label-free, data-driven framework for characterizing nanoparticle-induced structural changes in immune cells. The overall research workflow is illustrated in Figure 1.

## 2. Materials and Methods

### 2.1. Cell Culture and MNP Treatment

Mouse-derived RAW 264.7 macrophage cells were used to investigate morphological changes induced by MNP treatment. These cells were cultured in Dulbecco’s Modified Eagle Medium supplemented with 10% fetal bovine serum and 1% penicillin/streptomycin and incubated at 37 °C with 5% CO_2_ under standard humidified conditions [41]. To induce these changes, the MNPs employed were dextran-coated iron oxide particles (Micromod GmbH, Rostock, Germany), featuring an average particle size of 70 nm and carboxyl (–COOH) surface functionalization.

According to the manufacturer’s specifications and previously reported instrumental characterizations, dextran-coated iron oxide nanoparticles in this size range exhibit a narrow size distribution and stable colloidal behavior in aqueous media, as confirmed by dynamic light scattering (DLS) and transmission electron microscopy (TEM) analyses [18]. In addition, the presence of surface carboxyl groups results in a negative surface charge, which contributes to enhanced dispersion stability and facilitates efficient interaction and uptake by macrophages [21,22].

In the experimental group, macrophages were treated with MNPs at a concentration of 100 μg/mL for 4 h, while the control group was cultured under identical conditions without nanoparticle exposure. Following treatment, cellular images were acquired using a phase-contrast microscope (Eclipse Ti-U, Nikon Corporation, Tokyo, Japan) at 20× magnification and a resolution of 2560 × 1922 pixels. Notably, phase-contrast microscopy, as employed in this setup, provides a non-invasive and label-free method for visualizing live cell morphology, enabling real-time observation of structural changes triggered by external stimuli such as nanoparticle administration [42].

### 2.2. Image Acquisition and Preprocessing

Phase-contrast microscopy images were acquired from both the control and MNP-treated macrophage groups, yielding a dataset of approximately 2000 single-cell images. The raw images were manually cropped to isolate individual cells and subsequently resized to 256 × 256 pixels. To enhance structural clarity, each image was converted to grayscale, and CLAHE was applied to improve local contrast without over-amplification. A sharpening filter was then applied to enhance cellular boundaries and internal structures. Finally, pixel intensities were normalized to the 0–1 range to ensure stability and consistency during model training.

### 2.3. Model Architectures

**Figure 2:**
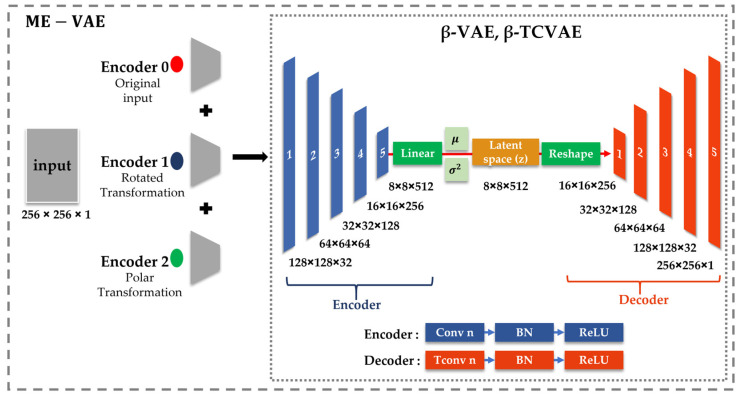
Overall architectures of the VAE-based models used in this study, namely β-VAE, β-total correlation VAE (TCVAE), and ME-VAE.

To analyze macrophage morphological variations induced by MNP treatment, three unsupervised learning frameworks based on VAEs were employed: β-VAE, β-TCVAE, and ME-VAE. The overall architectures of these VAE-based models are presented in Figure 2. These models were selected because they enable the extraction of latent space representations without manual labeling, thus facilitating an unbiased structural interpretation of cell morphology.

#### 2.3.1. β-VAE Model

The β-VAE framework introduces a weighting factor β into the Kullback–Leibler (KL) divergence term of the VAE loss function to encourage disentanglement among latent variables. Thus, by promoting independence among latent dimensions, the β-VAE model enhances structural separability, which is particularly beneficial for distinguishing subtle morphological differences between the control and MNP-treated cells. The corresponding loss function is defined as follows:

(1) $$L_{\beta -VAE}=E_{q(z|x)}\left[\log p(x|z)\right]-\beta \cdot D_{KL}\left(q(z|x\right)||p\left(z\right)),$$

where the first term corresponds to the reconstruction loss, while the second term regularizes the posterior distribution *q*(*z|x*) toward the prior *p*(*z*). Increasing β promotes disentanglement by penalizing correlations among latent variables.

#### 2.3.2. β-TCVAE Model

The β-TCVAE model extends the β-VAE framework by decomposing the KL divergence into three components: mutual information (MI), total correlation (TC), and dimension-wise KL divergence. By explicitly regulating the TC term, the β-TCVAE model reduces redundancy among latent variables and promotes more disentangled representations. This facilitates the clearer identification of distinct morphological features embedded within complex cellular images. The corresponding loss function is defined as

(2) $$L_{\beta -TCVAE}=E_{q(z|x)}\log\left[p(x|z)\right]-\alpha \cdot MI\left(x;z\right)-\beta \cdot TC\left(z\right)-\gamma \cdot \sum_{j}D_{KL}(q(z_{j})||p\left(z_{j}\right)),$$

where *MI*(*x*; *z*) represents the MI between inputs and latent variables, *TC(z)* captures dependencies among latent dimensions, and the final term denotes the dimension-wise KL divergence. By directly constraining the TC term via the parameter β, the model promotes independence among latent dimensions, thereby enhancing the interpretability of latent representations in complex cellular morphologies.

#### 2.3.3. ME-VAE Model

To capture morphological variability from multiple perspectives, the ME-VAE framework incorporates a multi-encoder architecture, wherein original, rotated, and polar-transformed images are concurrently processed by parallel encoders. Each encoder generates a latent vector *z*_i_, and the resulting vectors are integrated into a unified latent representation by averaging:

(3) $$z=\frac{1}{3}\left(z_{1}+z_{2}+z_{3}\right).$$

The aggregated latent vector is subsequently passed to the decoder to reconstruct the input image. The model employs the same β-VAE loss formulation, with β fixed at 6.0 in this study. By incorporating multiple augmented views, the ME-VAE enhances sensitivity to subtle morphological alterations, including membrane expansion, central density variation, and shape distortion. The overall learning process of the model is summarized in Algorithm 1.
**Algorithm 1:** Learning process of the multi-encoder variational autoencoder (ME-VAE) model

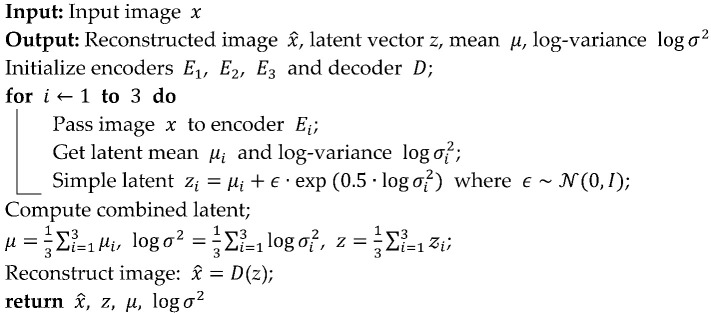


Collectively, these VAE-based models offer complementary strategies for uncovering the morphological responses of macrophages to nanoparticle treatment, yielding both quantitative and qualitative insights into latent feature representations.

### 2.4. Training Settings and Data Augmentation

All models were implemented using PyTorch 2.3.1 and trained with identical hyperparameters to ensure comparability. The dataset comprised 2000 single-cell images (1000 control and 1000 MNP-treated), each resized to 256 × 256 grayscale and preprocessed with contrast enhancement and normalization. The models were trained using the Adam optimizer with a learning rate of 1 × 10^−4^, a weight decay of 1 × 10^−5^, a batch size of 16, and 150 epochs. Mean squared error (MSE) was employed as the reconstruction loss, while KL-divergence-based regularization terms were applied depending on the model. For the ME-VAE specifically, data augmentation was applied to enhance morphological diversity. Each original image was augmented using two transformations:

1. Random rotations between −180° and +180°;
2. Polar flips derived from horizontal and vertical reflections.

The three image variants (original, rotated, and polar-transformed) were processed in parallel and integrated into a unified latent vector. Latent dimensionality was set to 32 for the β-VAE and β-TCVAE, whereas the ME-VAE was evaluated using both 32 and 64 dimensions. The primary training configurations are summarized in Table 1.

### 2.5. Evaluation Methods

The latent vectors generated by each VAE-based model were analyzed to characterize morphological differences between the control and MNP-treated groups. Three complementary evaluation methods were employed to extract both quantitative and qualitative insights.

#### 2.5.1. Cohen’s d Effect Size Analysis

Given that both groups comprised equal sample sizes (*n* = 1000), differences in latent distributions were quantified using Cohen’s *d* effect size [43,44]:

(4) $$d_{i}=\;\frac{\mu_{2,i}-\mu_{1,i}}{s_{p}},\;{\;s}_{p}=\;\sqrt{\frac{s_{1,i}^{2}+s_{2,i}^{2}}{2}},$$

where *d*_i_ denotes the effect size in the *i*th latent dimension; *μ*_1,i_ and *μ*_2,i_ are the group means; and *s*_1,i_ and *s*_2,i_ are the corresponding standard deviations for the control and MNP-treated groups, respectively. Latent dimensions with larger |*d*| values were considered to encode greater morphological differences.

#### 2.5.2. Kernel Density Estimation (KDE) Analysis

KDE was applied to the highest-ranked latent dimensions, based on effect size, to visualize intergroup distribution differences. Without assuming normality, KDE generates continuous density curves that facilitate direct visual comparison of shifts, overlaps, and separations between the control and MNP-treated groups in latent space [45,46].

#### 2.5.3. Latent Traversal and Difference Mapping

To interpret the morphological characteristics encoded in specific latent dimensions, each latent variable was linearly varied from −3 to +3, and the corresponding images were decoded. To quantify the resulting structural differences, two complementary visualization techniques were applied:

Absolute difference map: Pixel-wise absolute differences between reconstructed images generated at latent values of −3 and +3 [47].

SSIM-based difference mapping: SSIM-based mapping used to highlight structural discrepancies, with thresholding applied to emphasize regions of highest variation [40].

Collectively, these evaluation methods established a comprehensive framework to identify, quantify, and visualize MNP-induced morphological changes in macrophages within the latent space.

## 3. Results

### 3.1. Effect Size Analysis

**Figure 3:**
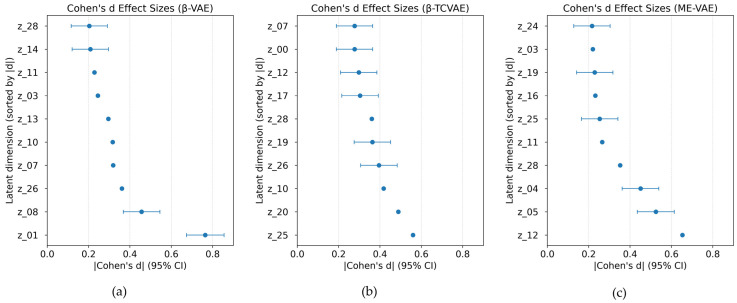
Forest plots of Cohen’s *d* effect sizes across latent dimensions for control and magnetic-nanoparticle (MNP)-treated macrophages: the (**a**) β-VAE, (**b**) β-TCVAE, and (**c**) ME-VAE. Error bars represent 95% confidence intervals.

Cohen’s *d* effect size was calculated for each latent dimension in the three VAE-based models to quantify morphological differences between the control and MNP-treated macrophages. Notably, the β-VAE and the β-TCVAE exhibited a limited number of latent dimensions with comparatively large effect sizes, indicating that morphological differences were concentrated in specific latent factors (Figure 3). In contrast, the ME-VAE demonstrated broader separation across multiple dimensions, suggesting that its multi-encoder design improved sensitivity to subtle morphological variations. These findings indicate that while the β-VAE and the β-TCVAE emphasize the disentanglement of specific latent variables, the ME-VAE distributes morphological information across a broader range of dimensions.

### 3.2. KDE Analysis

**Figure 4:**
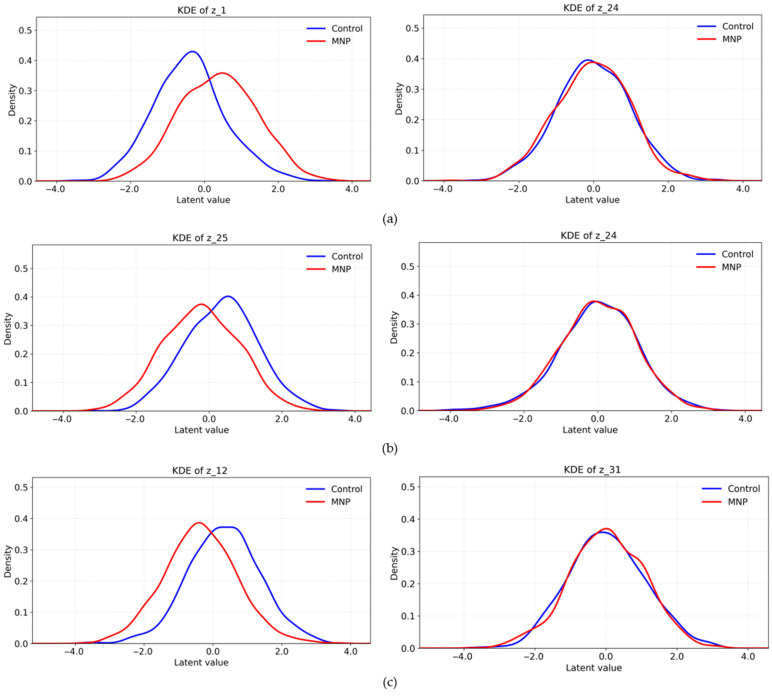
Kernel density estimation plots of representative latent dimensions for the control and MNP-treated macrophages: the (**a**) β-VAE, (**b**) β-TCVAE, and (**c**) ME-VAE.

Notably, the β-VAE incorporates a weighting factor, β, into the KL divergence term to promote disentanglement of latent variables. In this study, the model was trained using β = 6.0 and a latent dimension of 32. KDE analysis revealed that the top-ranked dimension (*z*_1_) exhibited clear separation between the control and MNP-treated groups (Figure 4a). The control group exhibited a broader, more symmetric distribution, while the MNP-treated group was skewed toward higher values with a sharper peak, indicating a distinct morphological alteration. In contrast, the low-effect dimension (*z*_24_) displayed largely overlapping distributions, suggesting limited capacity for morphological discrimination. These findings demonstrate that the β-VAE selectively captures MNP-induced morphological changes in specific latent dimensions.

In parallel, the β-TCVAE decomposes the KL divergence into MI, TC, and dimension-wise components to reduce redundancy among latent variables. In this study, the β-TCVAE model was trained with β = 6.0 and a latent dimension of 32. KDE analysis revealed that the top-ranked dimension (z_25_) exhibited clear separation, with the MNP-treated group displaying a sharper distribution shifted toward higher values and the control group exhibiting a flatter and broader curve (Figure 4b). In contrast, the low-effect dimension (z_24_) displayed nearly identical distributions between groups, confirming its limited discriminative contribution. These findings suggest that the β-TCVAE produces disentangled representations that facilitate clearer group separation in specific latent dimensions. Finally, the ME-VAE employs a multi-encoder design that integrates original, rotated, and polar-transformed images into a unified latent representation. The model was trained using β = 6.0 and a latent dimension of 32. KDE analysis revealed that the top-ranked dimension (z_12_) exhibited the most pronounced separation (Figure 4c). The MNP-treated group shifted toward the left side of the axis, forming a dense and sharp peak, whereas the control group displayed a broader and more uniform distribution. In contrast, the low-effect dimension (z_31_) displayed heavily overlapping distributions, indicating minimal contribution to group discrimination. These results indicate that the ME-VAE, by integrating multiple visual perspectives, is more sensitive to subtle MNP-induced morphological variations than the other models.

### 3.3. Violin Plot Analysis

**Figure 5:**
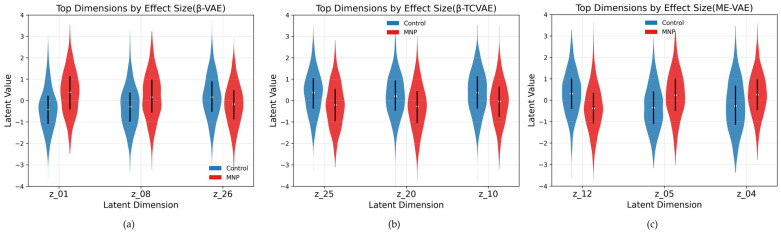
Violin plots of the top latent dimensions ranked by Cohen’s d effect size for the control and MNP-treated macrophages in the (**a**) β-VAE, (**b**) β-TCVAE, and (**c**) ME-VAE. White dots denote medians, black bars indicate interquartile ranges, and the width of each shape reflects the distribution density.

To further examine the latent dimensions with the largest effect sizes, violin plots were generated for the top three dimensions identified in each model (Figure 5). These plots illustrate both the central tendency and overall distribution profiles of the control and MNP-treated groups. In the β-VAE, dimensions z_1_, z_8_, and z_26_ exhibited clear distributional separation between the two groups (Figure 5a). Notably, z_1_ revealed a pronounced shift in the central density of the MNP-treated group toward higher values, whereas z_8_ and z_26_ exhibited variations in spread, suggesting increased morphological heterogeneity following MNP treatment. In the β-TCVAE, dimensions z_25_, z_20_, and z_10_ demonstrated consistent divergence in both mean position and distributional range (Figure 5b). Specifically, z_25_ showed a shift in the MNP-treated group toward lower values, accompanied by a broader distribution that reflected greater variability. Dimensions z_20_ and z_10_ further confirmed that MNP-induced alterations involved not only mean shifts but also distributional broadening, indicating cell-to-cell variability in morphological response. In the ME-VAE, top-ranked dimensions z_12_, z_5_, and z_4_ exhibited distinctive bimodal-like patterns in the MNP-treated group relative to the more compact control distributions (Figure 5c). This suggests that the ME-VAE captured nuanced morphological subpopulations that emerged following MNP treatment. Notably, z_12_ exhibited a marked peak shift in density, whereas z_4_ displayed broader tails, indicating structural differences less prominently captured by the other models. Overall, violin plot analysis offered complementary insights beyond those provided by the forest and KDE plots by capturing both central tendency shifts and distributional variability, thereby reinforcing the ability of the VAE-based frameworks to disentangle the heterogeneous morphological responses of macrophages to nanoparticle treatment.

### 3.4. Traversal-Based Morphological Analysis

**Figure 6:**
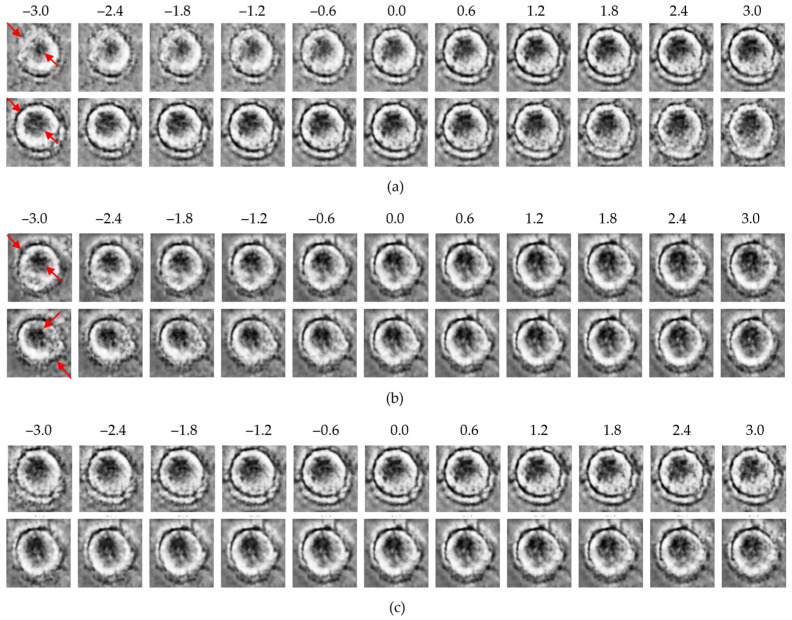
Latent traversal results from the β-VAE model: (**a**) control group traversals along the top-ranked latent dimensions (*z*_1_, upper row; *z*_8_, lower row); (**b**) MNP-treated group traversals along the same dimensions (*z*_1_, upper row; *z*_8_, lower row); (**c**) traversal along the lowest-ranked dimension (*z*_24_; upper: control, lower: MNP). Red arrows indicate regions with the most significant morphological variations observed during the latent space traversal.

**Figure 7:**
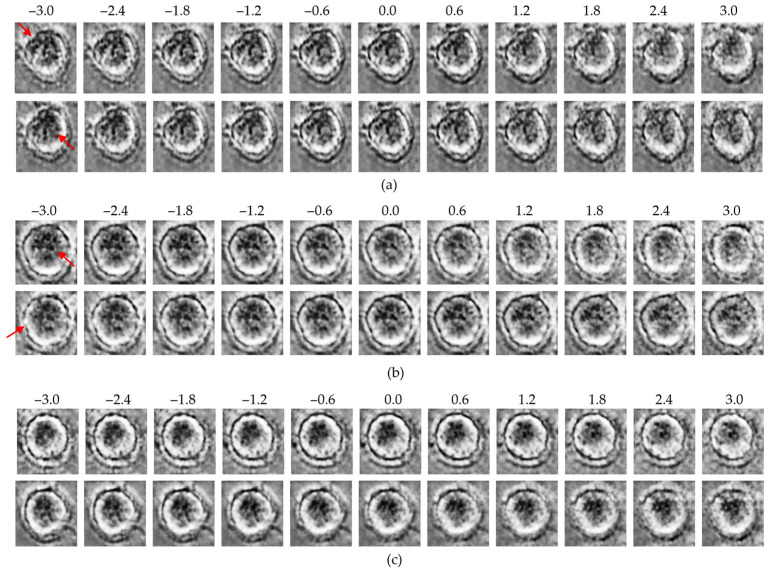
Latent traversal results from the β-TCVAE model: (**a**) control group traversals along high-effect latent dimensions (*z*_25_, upper row; *z*_20_, lower row); (**b**) MNP-treated group traversals along the same dimensions (*z*_25_, upper row; *z*_20_, lower row); (**c**) traversal along the low-effect dimension (*z*_24_; upper: control, lower: MNP). Red arrows indicate regions with the most significant morphological variations observed during the latent space traversal.

**Figure 8:**
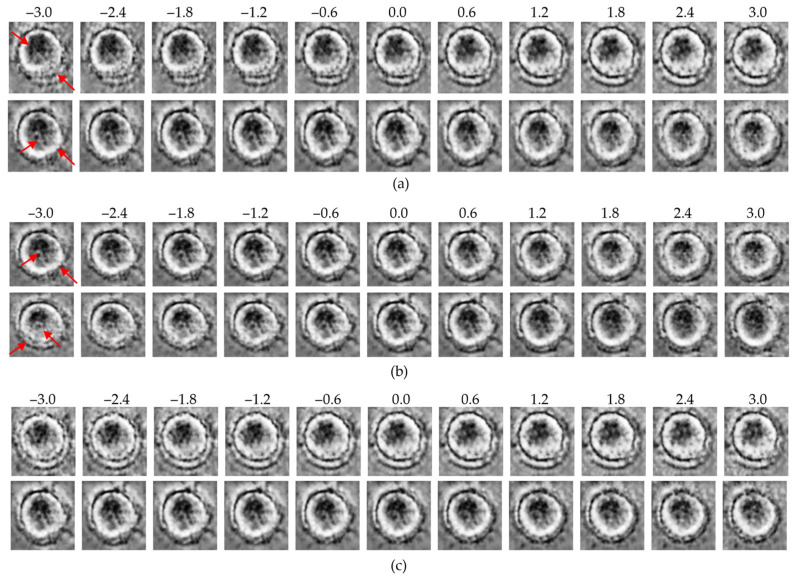
Latent traversal results from the ME-VAE model: (**a**) control group traversals along the top-ranked latent dimensions (*z*_12_, upper row; *z*_5_, lower row); (**b**) MNP-treated group traversals along the same dimensions (*z*_12_, upper row; *z*_5_, lower row); (**c**) traversal along the lowest-ranked dimension (*z*_31_; upper: control, lower: MNP). Red arrows indicate regions with the most significant morphological variations observed during the latent space traversal.

To qualitatively explore the latent representations, traversal experiments were conducted by systematically varying specific latent dimensions across a range of −3 to +3. This approach enabled direct visualization of how individual latent variables encode macrophage morphology and whether these encodings differ between the control and MNP-treated groups. Representative results for each model are presented in Figure 6, Figure 7 and Figure 8.

In all three models, traversals along high-effect latent dimensions revealed distinct structural variations. At the cell periphery, changes in density and boundary contrast were observed. Meanwhile, within the intracellular region, progressive modulations in intensity and texture reflected variability in cytoplasmic organization. These changes were more pronounced in the MNP-treated group, suggesting that high-effect dimensions encode biologically relevant features linked to nanoparticle-induced structural responses.

In contrast, traversal along low-effect dimensions resulted in minimal morphological changes, with cell structures remaining relatively stable across the full traversal range. These dimensions appear to capture background noise or redundant variance rather than biologically meaningful features.

Overall, the β-VAE, the β-TCVAE, and the ME-VAE consistently revealed that biologically informative features are concentrated within a subset of high-effect latent dimensions, whereas low-effect dimensions primarily encode uninformative variance. This finding highlights the common ability of the VAE-based models to disentangle and localize treatment-related morphological variations within specific latent factors.

### 3.5. Difference Map Analysis

**Figure 9:**
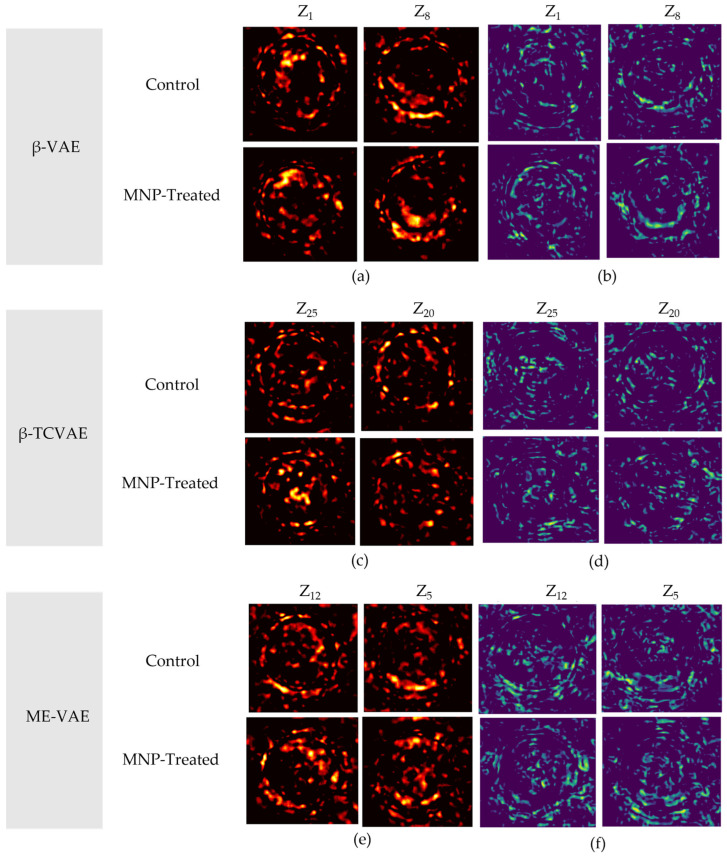
Difference map analysis results of the VAE-based models: (**a**) β-VAE absolute difference maps for top-ranked latent dimensions (z_1_, z_8_); (**b**) β-VAE structural-similarity-index-measure (SSIM)-based difference maps for the same dimensions; (**c**) β-TCVAE absolute difference maps for top-ranked latent dimensions (z_25_, z_20_); (**d**) β-TCVAE SSIM-based difference maps for the same dimensions; (**e**) ME-VAE absolute difference maps for top-ranked latent dimensions (z_12_, z_5_); (**f**) ME-VAE SSIM-based difference maps for the same dimensions.

**Figure A1:**
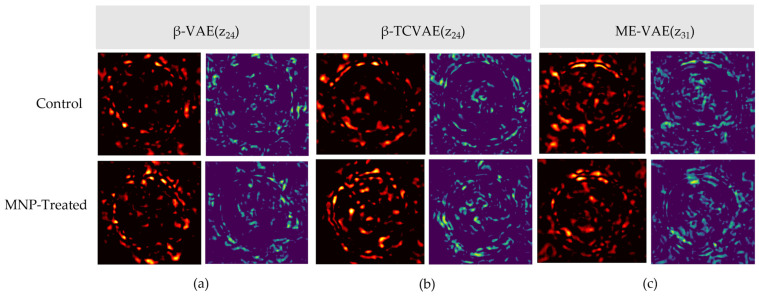
Difference map analysis for low-effect latent dimensions across the VAE-based models: the (**a**) β-VAE (z_24_), absolute (left) and SSIM (right) difference maps; (**b**) β-TCVAE (z_24_), absolute (left) and SSIM (right) difference maps; (**c**) ME-VAE (z_31_), absolute (left) and SSIM (right) difference maps.

**Figure A2:**
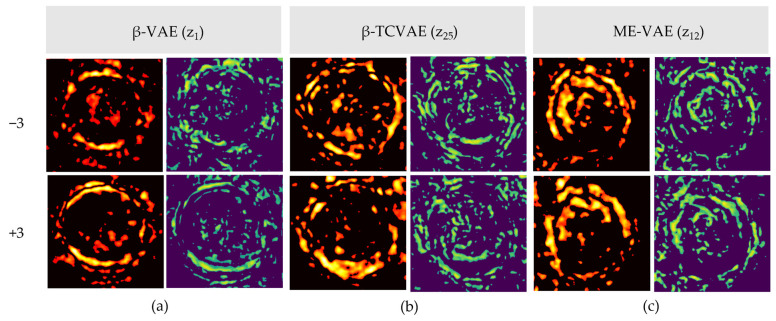
Comparison of control and MNP-treated reconstructions at equivalent latent values: the (**a**) β-VAE (z_1_), (**b**) β-TCVAE (z_25_), and (**c**) ME-VAE (z_12_).

Difference maps were generated by computing differences between reconstructed images at the latent extremes (−3 and +3), using both absolute pixel-wise differences and SSIM-based metrics (Figure 9). In all models, high-effect latent dimensions consistently localized morphological alterations to the cell periphery and cytoplasmic regions. Conversely, low-effect dimensions (e.g., z_24_) exhibited minimal structural variation, indicating that they primarily encode uninformative variance such as background noise or redundant features (Figure A1 of Appendix A.1). For completeness, difference maps comparing the control and MNP-treated groups at matched latent values are presented in Appendix A.2 (Figure A2).

In the β-VAE model, traversal along high-effect dimensions such as z_27_ revealed marked differences in boundary contrast and cytoplasmic density, corresponding to nanoparticle-induced membrane remodeling and cytoskeletal alterations. The absolute difference maps primarily emphasized localized intensity changes at the membrane, whereas the SSIM maps accentuated structural distortions, collectively demonstrating that these dimensions robustly encode biologically meaningful features. These findings align with actin cytoskeletal rearrangements and reactive-oxygen-species-mediated remodeling processes that restrict cell motility and reshape membrane curvature following nanoparticle exposure [48]. Moreover, the strong association between endocytic uptake and actin-driven invagination further supports the interpretation that these latent features reflect nanoparticle-induced membrane remodeling processes [49].

The β-TCVAE exhibited enhanced disentanglement capacity. Treatment-related alterations were identified across a broader range of latent dimensions, localizing changes to both the membrane and intracellular regions. This pattern is directly attributable to the model’s explicit suppression of redundancy through TC regularization, thereby enabling more precise identification of nanoparticle-induced responses. These features are consistent with biological evidence indicating that iron oxide nanoparticles activate TLR4-dependent signaling cascades, thereby promoting autophagic flux and inflammatory responses that drive cytoplasmic compartment reorganization [50].

In the ME-VAE, morphological differences were more broadly distributed across cellular regions. The integration of augmented input views allowed the model to detect both peripheral membrane expansion and cytoplasmic textural heterogeneity. This outcome is consistent with the endolysosomal accumulation of MNPs and the associated reorganization of intracellular compartments. Transmission electron microscopy studies have demonstrated that internalized iron oxide nanoparticles predominantly accumulate within endosomes and lysosomes, thereby altering cytoplasmic density and compromising compartmental integrity [51]. Furthermore, the surface chemistry of nanoparticles directly influences intracellular trafficking and degradation kinetics, thereby modulating endolysosomal remodeling pathways [52]. Collectively, these findings support the broader concept of nanoparticle-induced macrophage reprogramming, wherein endocytic processing, cytoskeletal adaptation, and intracellular reorganization jointly alter cellular phenotypes [53].

Together, these results demonstrate that difference map analysis yields spatially resolved evidence of MNP-induced morphological alterations. The observed structural variations correspond closely to established biological mechanisms, reinforcing the capacity of VAE-based frameworks to disentangle and localize biologically meaningful features of macrophage morphology in an unsupervised manner.

## 4. Discussion

In this study, we employed three unsupervised learning frameworks, namely the β-VAE, β-TCVAE, and ME-VAE, to investigate morphological alterations in macrophages following treatment with MNPs. In contrast to conventional nanoparticle studies that predominantly assess uptake efficiency or cytotoxicity as endpoint metrics, our work focused on revealing subtle, spatially distributed morphological remodeling through unsupervised representation learning. This approach offers a complementary perspective on nanoparticle–cell interactions that extends beyond traditional quantitative measures.

Each of these models demonstrated distinct capabilities in disentangling and localizing biologically informative features within the latent space. Specifically, the β-TCVAE demonstrated enhanced disentanglement by suppressing redundant variance and enabling clearer separation of treatment-related dimensions. In contrast, the ME-VAE, which employs a multi-encoder design with augmented inputs, exhibited superior sensitivity in detecting subtle morphological variations, capturing both peripheral and cytoplasmic alterations with improved spatial resolution. These findings underscore that architectural design choices critically influence how biologically relevant morphological information is encoded and interpreted within unsupervised latent spaces, highlighting the importance of model selection in capturing meaningful cellular features. Collectively, these findings suggest that the representation of biologically relevant features is strongly influenced by model architecture, highlighting the critical role of design choices in the morphological interpretation of cell imaging data.

Traversal and difference map analyses revealed that high-effect latent dimensions consistently encoded biologically informative morphological features, including membrane remodeling, cytoplasmic density modulation, and texture reorganization. These morphological changes are consistent with established cellular mechanisms of nanoparticle uptake, including cytoskeletal remodeling and endolysosomal accumulation. By establishing connections between latent-space variations and previously documented cellular mechanisms, our results demonstrate that unsupervised latent traversal can function as an interpretable framework bridging data-driven representations with biologically meaningful processes. This linkage facilitates mechanistic interpretation of emergent patterns in the latent space. Importantly, the reconstructions of the ME-VAE displayed membrane expansion and intracellular heterogeneity, which aligned with early manifestations of MNP-induced cellular remodeling. In contrast, low-effect dimensions across all models primarily encoded background variance or redundant features, underscoring the selective localization of biologically informative signals within the latent space.

Importantly, differences observed across model-specific latent representations reflect complementary sensitivities of each architecture rather than contradictions. Accordingly, aggregated trends are used to summarize consistent morphological patterns across models, while model-specific effect sizes are retained to preserve interpretability and transparency.

Despite these insights, several limitations must be acknowledged. First, the analysis was restricted to a single macrophage cell line and a fixed imaging condition (phase-contrast microscopy at 256 × 256 resolution), which limits the generalizability of the findings to other cell types and imaging modalities. Second, the interpretations were primarily qualitative, as the models were trained without explicit biological labels. Although the traversal and difference maps yielded valuable insights, biological validation remains essential.

While conventional manual morphology metrics provide intuitive and interpretable descriptors, they are inherently limited to predefined features and may not fully capture the subtle, spatially distributed remodeling patterns addressed by our unsupervised framework. Importantly, the proposed framework exhibits broad applicability beyond macrophages and magnetic nanoparticles, being readily adaptable to diverse cell types, nanomaterials, and imaging modalities. This versatility establishes a generalizable methodology for label-free morphological analysis under live-cell imaging conditions. Incorporating simple morphological indices, such as nuclear-to-cytoplasmic ratio, cell size measurements, or texture-based quantification, would substantially strengthen the biological relevance of the findings. Future studies should also explore multi-cell-type experiments, diverse treatment conditions, and higher-resolution imaging modalities (e.g., fluorescence or confocal microscopy) to enhance robustness, biological interpretability, and translational value.

## 5. Conclusions

This study compared the β-VAE, β-TCVAE, and ME-VAE models for unsupervised analysis of macrophage morphology following MNP treatment. Through a combination of Cohen’s *d* effect size analysis, KDE, traversal-based visualization, and difference map analysis, all three frameworks were demonstrated to capture treatment-induced morphological alterations with differing degrees of latent disentanglement and spatial specificity. Among the tested models, the β-TCVAE consistently disentangled treatment-sensitive latent dimensions, while the ME-VAE exhibited enhanced sensitivity to spatially distributed morphological responses through the integration of augmented perspectives.

The observed morphological alterations, including shifts in membrane density, cytoplasmic organization, and intensity distribution, align with established biological processes such as cytoskeletal remodeling and endolysosomal reorganization. These findings underscore the potential of VAE-based models as unsupervised tools for identifying label-free signatures of nanoparticle–cell interactions.

Future studies should expand these analyses to include diverse cell types and time-course conditions and employ higher-resolution imaging techniques to capture finer subcellular structures. Moreover, linking latent variables to quantifiable biological indices will improve the interpretability of unsupervised learning frameworks and support their application in digital pathology and nanomedicine.

## Figures and Tables

**Table 1:** Key training configurations for the β-VAE, β-TCVAE, and ME-VAE.

Model	Latent Dimension	β Regularization Term	Total Correlation	Data Augmentation
β-VAE	32	KL divergence	×	None
β-TCVAE	32	TC term ( α=1,β=6,γ=1)	○	None
ME-VAE	32, 64	KL divergence	×	Rotation; Polar flip

## Data Availability

The original contributions presented in this study are included in the article. Further inquiries can be directed to the corresponding author(s).

## Appendix A

### Appendix A.1. Additional Difference Map Results for Low-Effect Latent Dimensions

To extend the main findings, supplementary difference map analyses were conducted for low-effect latent dimensions. These maps were generated by subtracting reconstructed images at latent extremes (−3 vs. +3) and were evaluated using absolute pixel-wise differences and SSIM metrics.

As illustrated in Figure A1, the low-effect latent dimensions displayed negligible morphological variation in both the control and MNP-treated groups. The absolute maps showed slight intensity shifts at the cell periphery, whereas the SSIM maps confirmed high structural similarity across reconstructions. These findings support the interpretation that such dimensions primarily encode background noise or redundant variance, rather than biologically meaningful morphological features.

### Appendix A.2. Control vs. MNP-Treated Difference Maps at Equivalent Latent Values

To further assess treatment-specific morphological changes, reconstructed images from the control and MNP-treated groups were compared at equivalent latent values (−3 and +3). Representative comparisons are presented in Figure A2. In the high-effect latent dimensions (e.g., z_27_ in the β-VAE, z_25_ in the β-TCVAE, and z_12_ in the ME-VAE), group comparisons revealed consistent structural alterations at the cell membrane and within cytoplasmic compartments, consistent with nanoparticle-induced remodeling and intracellular reorganization. Notably, owing to intrinsic variability in cell size and shape among individual cells, these maps are not intended to display precise pixel-wise differences but rather to illustrate generalized morphological patterns localized within biologically relevant areas.
